# Nasopharyngeal tuberculosis mimicking malignancy on MRI: A rare clinical entity in an immunocompetent adult

**DOI:** 10.1016/j.radcr.2026.04.088

**Published:** 2026-06-03

**Authors:** Mohamed Bouallou, Issam Berrajaa, Achraf Amine Sbai, Drissia Benfadil, Azeddine Lachkar

**Affiliations:** a Faculty of Medicine and Pharmacy, Mohammed First University, Oujda 60000, Morocco; b Department of Otorhinolaryngology, Mohammed VI University Hospital, Oujda 60000, Morocco; c Laboratory of Oto-Neuro-Ophthalmology (LORNO), Faculty of Medicine and Pharmacy, Mohammed First University, Oujda 60000, Morocco; d Faculty of Medicine and Pharmacy, Mohammed First University, LAMCESM, Oujda 60000, Morocco

**Keywords:** Nasopharyngeal tuberculosis, Extrapulmonary tuberculosis, Magnetic resonance imaging, Nasopharyngeal mass, Malignancy

## Abstract

Nasopharyngeal tuberculosis (NPTB) represents an uncommon manifestation of extrapulmonary tuberculosis and may closely simulate malignant disease on magnetic resonance imaging, particularly when it exhibits aggressive features. Its rarity and nonspecific clinical presentation often lead to a significant diagnostic challenge. We report the case of a 41-year-old immunocompetent woman presenting with chronic right-sided headaches and hearing loss, in whom MRI revealed a large infiltrative mass arising from the right posterolateral wall of the nasopharynx with extension to adjacent deep spaces, raising strong suspicion of malignancy. Histopathological examination of biopsy specimens demonstrated necrotizing granulomatous inflammation with central caseous necrosis, and PCR testing confirmed infection with *Mycobacterium tuberculosis*. The patient was treated with standard antituberculous therapy, resulting in complete clinical and radiological resolution. This case highlights that nasopharyngeal tuberculosis should be considered in the differential diagnosis of aggressive nasopharyngeal masses, even in immunocompetent patients without systemic symptoms, and underscores the essential role of histopathological confirmation.

## Introduction

Tuberculosis is a chronic granulomatous infectious disease caused by *Mycobacterium tuberculosis* and remains a major global public health concern. Although pulmonary involvement is the most common manifestation, extrapulmonary tuberculosis accounts for a significant proportion of cases, particularly in endemic regions. Involvement of the upper aerodigestive tract is uncommon, representing less than 1% of extrapulmonary cases, with the nasopharynx being an exceptionally rare site [1].

Nasopharyngeal tuberculosis is an exceptionally rare form of extrapulmonary tuberculosis, even in tuberculosis-endemic regions such as Morocco [2]. It may occur as a primary infection but more commonly represents a secondary localization related to pulmonary tuberculosis, resulting from direct contamination of the nasopharyngeal mucosa by infected bronchopulmonary secretions [3]. Owing to its rarity and nonspecific clinical presentation, NPTB poses a considerable diagnostic challenge [4].

From a radiological perspective, NPTB may closely mimic malignant tumors of the nasopharynx, particularly nasopharyngeal carcinoma or lymphoma. MRI, the modality of choice for evaluating nasopharyngeal lesions, may reveal infiltrative masses with aggressive features, further complicating the distinction between infectious and malignant etiologies [5]. Consequently, imaging findings alone are insufficient for diagnosis, and histopathological confirmation remains essential.

We report a rare case of primary nasopharyngeal tuberculosis in an immunocompetent adult, presenting with chronic headaches and an aggressive-appearing nasopharyngeal mass on MRI, initially suggestive of malignancy. This case highlights the diagnostic challenges of NPTB and underscores the importance of including this entity in the differential diagnosis of nasopharyngeal masses.

## Case presentation

A 41-year-old Moroccan woman with a history of intellectual disability and no known medical or surgical comorbidities was referred to our Department of Otorhinolaryngology for evaluation of persistent headaches evolving over a 7-month period. The headaches were predominantly right-sided and retro-orbital, with progressive worsening during the last 2 months prior to presentation. In addition, the patient reported right-sided hearing loss that had developed 3 months earlier.

She had been treated with step III analgesics for approximately 3 months without clinical improvement, prompting neurological evaluation during the seventh month of symptom progression, followed by referral to our otorhinolaryngology department for further assessment.

The patient denied fever, night sweats, weight loss, or any previous history of tuberculosis. There were no known immunosuppressive conditions, and she was not receiving any long-term medications.

On general examination, the patient was conscious and clinically stable, with normal respiratory and hemodynamic parameters. Flexible nasopharyngoscopy demonstrated normal-appearing nasal mucosa without turbinate hypertrophy or choanal obstruction. A right-sided exophytic, non-bleeding mass arising from the lateral wall of the nasopharynx was identified, with no bleeding on contact (Fig. 1). Otoscopic examination revealed a normal external auditory canal and an intact tympanic membrane, associated with right-sided serous otitis media. No palpable cervical lymphadenopathy was detected.

**Fig. 1:**
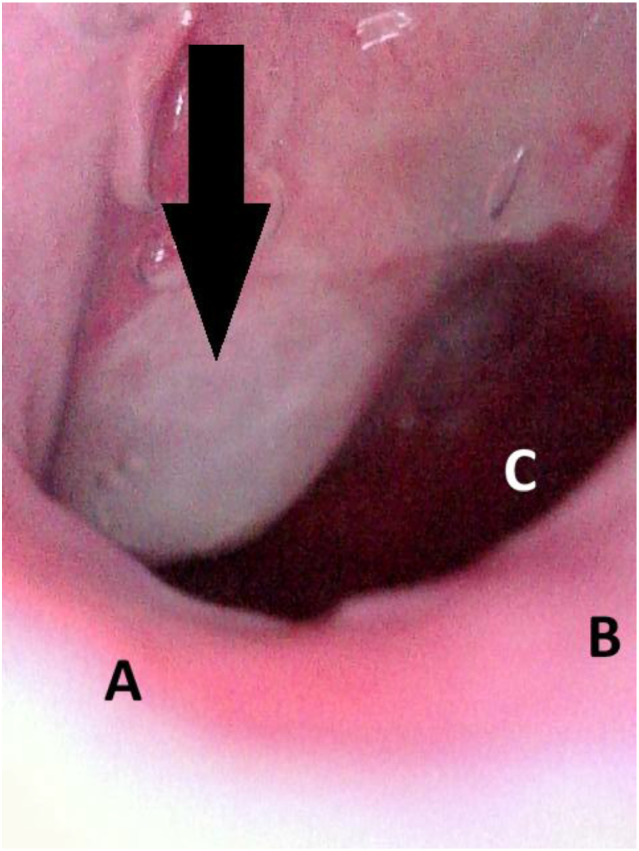
Endoscopic view of the nasopharynx demonstrating a right-sided exophytic mass arising from the lateral wall of the nasopharynx (black arrow). (A) Floor of the right nasal cavity; (B) Nasal septum; (C) Pharyngeal orifice of the Eustachian tube.

Pure-tone audiometry demonstrated a right-sided conductive hearing loss, with an average air–bone gap corresponding to a mean hearing loss of 39 dB.

Laboratory investigations revealed a hemoglobin level of 15.3 g/dL and an elevated white blood cell count of 12,700/mm³. Inflammatory markers were increased, with a C-reactive protein level of 29 mg/L and an erythrocyte sedimentation rate of 60 mm/h. Liver and renal function tests, serum electrolyte levels, coagulation parameters, and glycated hemoglobin (HbA1c: 5.3%) were within normal limits. Serological testing for human immunodeficiency virus (HIV) was negative.

Contrast-enhanced magnetic resonance imaging was performed on a 1.5-T system. The imaging protocol included axial and coronal T1-weighted sequences (slice thickness: 4 mm), axial and coronal T2-weighted sequences, fat-suppressed T2-weighted images, diffusion-weighted imaging (DWI) with b-values of 0 and 1000 s/mm², with corresponding apparent diffusion coefficient (ADC) maps, and post-contrast fat-suppressed T1-weighted sequences following intravenous administration of a gadolinium-based contrast agent (0.1 mmol/kg). The imaging field of view included the nasopharynx and the cervical region.

MRI demonstrated a large infiltrative mass involving the right posterolateral wall of the nasopharynx. The lesion exhibited irregular and poorly defined margins, was isointense on T1-weighted images and showed intermediate signal intensity on T2-weighted images, without evidence of central necrosis, with marked hyperintensity on diffusion-weighted imaging (b = 1000 s/mm²), consistent with diffusion restriction. However, quantitative ADC values were not available in this case. The lesion demonstrated heterogeneous enhancement following gadolinium administration. The mass measured approximately 45 × 35 mm and extended into the right parapharyngeal fat and infratemporal fossa, with encasement of the right carotid–jugular vascular bundle. Although the lesion extended superiorly toward the skull base, no clival marrow signal abnormality, skull base foraminal involvement, perineural spread, or dural enhancement was identified, and there was no evidence of intracranial extension. No cervical or retropharyngeal lymphadenopathy was identified on imaging, and no necrotic lymph nodes were detected (Fig. 2). Chest radiography revealed no abnormalities.

**Fig. 2:**
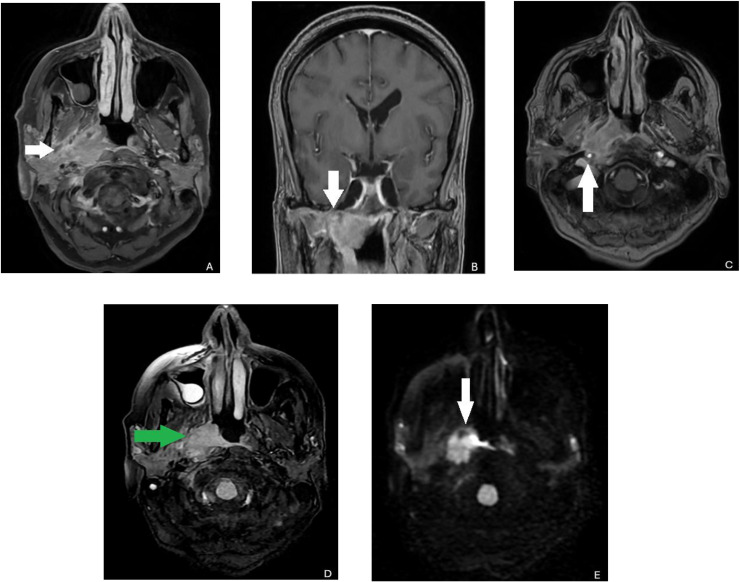
Magnetic resonance imaging findings of the nasopharyngeal lesion**.** (A) Axial contrast-enhanced T1-weighted image demonstrating a large, heterogeneously enhancing mass arising from the right posterolateral wall of the nasopharynx, extending to the infratemporal fossa (white arrow). (B) Coronal contrast-enhanced T1-weighted image showing superior extension of the lesion toward the skull base, without evidence of intracranial or brain parenchymal invasion (white arrow). (C) Axial T2-weighted image revealing a mass of intermediate signal intensity abutting the right carotid space and extending into the parapharyngeal space (white arrow). (D) Axial fat-suppressed T2-weighted image confirming the intermediate signal intensity of the lesion (green arrow). (E) Diffusion-weighted imaging (DWI) demonstrating marked hyperintensity of the mass (white arrow), consistent with diffusion restriction.

A biopsy of the nasopharyngeal lesion was performed using a rigid 30-degree endoscope. Histopathological examination demonstrated necrotizing granulomatous inflammation composed of epithelioid histiocytes and multinucleated giant cells, associated with central caseous necrosis (Fig. 3). Nested TB-PCR testing was positive, confirming the diagnosis of nasopharyngeal tuberculosis.

**Fig. 3:**
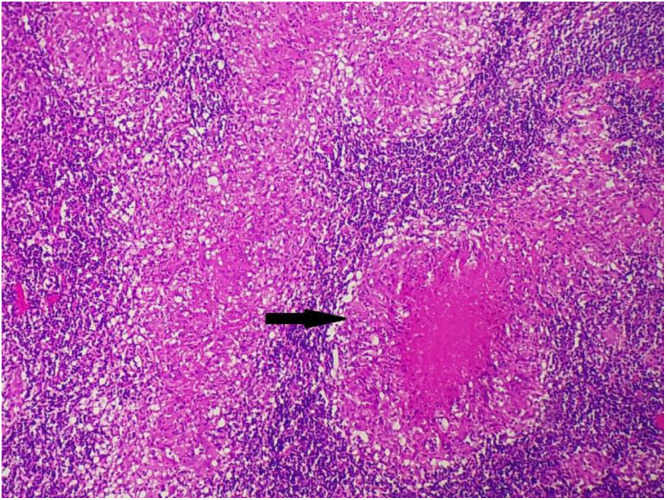
Histopathological section demonstrating central caseous necrosis surrounded by epithelioid cell granulomas with multinucleated giant cells (black arrow) (hematoxylin and eosin stain).

The patient was started on standard antituberculous therapy consisting of a 2-month intensive phase with rifampicin, isoniazid, pyrazinamide, and ethambutol, followed by a 4-month continuation phase with rifampicin and isoniazid (2RHZE/4RH). In parallel, a transtympanic ventilation tube (T-tube) was inserted in the right ear for management of the serous otitis media.

Treatment was well tolerated, with no reported adverse effects. Monthly follow-up included a comprehensive otorhinolaryngological examination with flexible nasopharyngoscopy, cervical assessment, chest radiography, and monitoring of liver function tests. A computed tomography scan performed at 5 months demonstrated complete radiological resolution of the nasopharyngeal mass (Fig. 4).

**Fig. 4:**
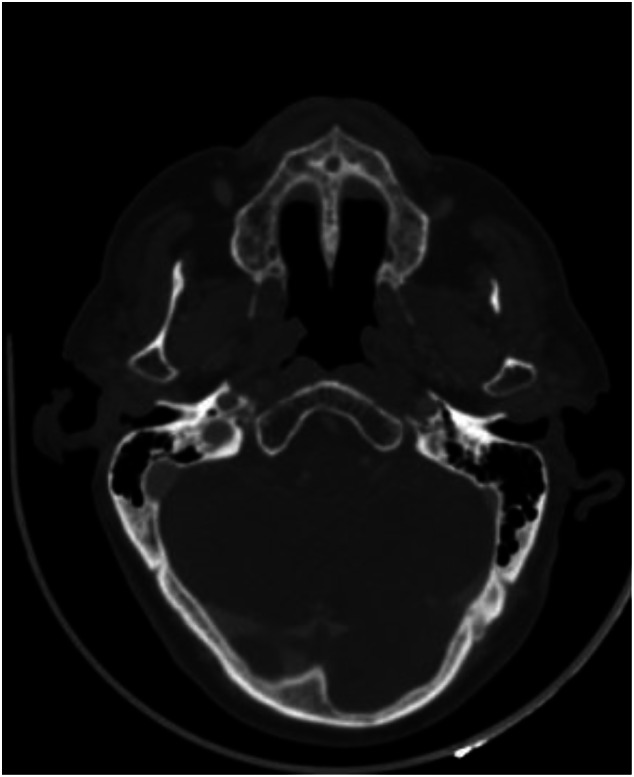
Axial CT scan of the head and neck demonstrating complete resolution of the previously identified nasopharyngeal mass.

Clinical evolution was favorable. At 9 months of follow-up, the patient remained asymptomatic, with no clinical or endoscopic evidence of disease recurrence.

## Discussion

NPTB, first described by Morgagni in 1761, is an exceptionally rare form of extrapulmonary tuberculosis, accounting for less than 1% of tuberculosis cases involving the upper aerodigestive tract [6]. Even in regions where tuberculosis is endemic, nasopharyngeal involvement remains uncommon and is likely underdiagnosed owing to the paucity and nonspecific nature of its clinical manifestations [2]. Primary NPTB is considered an exceptionally rare entity, with the existing literature largely limited to isolated case reports [7]. Consequently, NPTB represents a significant diagnostic challenge for both otolaryngologists and radiologists, particularly in its late clinical stages.

Two principal pathogenic mechanisms have been described in NPTB: direct mucosal inoculation of *M tuberculosis*, favored by the relative absence of effective mucociliary clearance, and secondary hematogenous or lymphatic dissemination facilitated by the rich lymphoid and vascular network of the nasopharynx [8]. In our patient, the history of unpasteurized milk consumption and the absence of initial cervical lymphadenopathy support a primary nasopharyngeal infection via direct contamination.

Clinically, NPTB often manifests with nonspecific local symptoms and frequently lacks constitutional features such as weight loss, night sweats, or respiratory complaints [9]. In addition, the complex anatomy of the nasopharynx combined with this nonspecific clinical presentation contributes to significant diagnostic difficulty.

In our case, the clinical presentation was particularly atypical, as the patient presented exclusively with chronic headaches. Notably, there was no initial cervical lymphadenopathy, despite it being the most frequently reported clinical manifestation, nor were there nasal obstruction or epistaxis [10].

Several atypical clinical presentations of nasopharyngeal tuberculosis have been reported in the literature. Özcan et al. [11] described a 17-year-old female presenting with cervical swelling and hearing loss, while Nakao et al. [7] reported a case manifesting primarily with posterior nasal drip and chronic cough.

The unusual presentation in our case further compounded the diagnostic challenge and highlights the highly variable and often misleading clinical spectrum of nasopharyngeal tuberculosis, underscoring the need to maintain a high index of suspicion even in the absence of classic symptoms.

Imaging data regarding nasopharyngeal tuberculosis remain scarce and are predominantly limited to isolated case reports. MRI plays a pivotal role in the assessment of nasopharyngeal lesions by enabling detailed evaluation of lesion morphology, signal characteristics, and patterns of local extension. In malignant entities, particularly nasopharyngeal carcinoma and non-Hodgkin lymphoma, invasion of adjacent deep structures is a frequent imaging feature and represents a key criterion of aggressiveness [12].

In contrast, NPTB typically demonstrates a more superficial pattern of involvement, with disease confined to the mucosal and submucosal layers of the nasopharynx [8]. In a retrospective MRI-based study by Cai et al. [12] including 21 patients, all lesions were limited to the nasopharyngeal mucosa without extension to adjacent structures, a finding corroborated by several other reports describing localized disease without parapharyngeal involvement [13,14]. Nevertheless, rare cases of deeper extension have been reported, such as involvement of the longus capitis muscle described by Percodani et al. [15].

Accordingly, extension to deep adjacent structures is generally more suggestive of malignancy, particularly undifferentiated carcinoma of the nasopharynx [16]. However, certain imaging features, such as the presence of central necrosis within the lesion and predominant involvement of the nasopharyngeal walls, may favor a tuberculous etiology over carcinoma [8]. In the present case, the lesion was located along the posterolateral wall of the nasopharynx. Nevertheless, MRI did not demonstrate central necrosis, nor was there associated cervical lymphadenopathy, rendering the diagnosis of NPTB particularly challenging based on imaging findings alone.

In the absence of cervical lymphadenopathy, a broad spectrum of differential diagnoses must be considered when interpreting aggressive nasopharyngeal lesions on MRI. These include malignant entities such as rhabdomyosarcoma, lymphoma, and extramedullary plasmacytoma, as well as inflammatory and granulomatous diseases including sarcoidosis, granulomatosis with polyangiitis (Wegener’s granulomatosis), fungal infections, and syphilis. Many of these conditions may present as infiltrative nasopharyngeal masses with imaging features that closely mimic malignancy.

In this clinical context, a structured and systematic radiological differential diagnosis is essential, particularly in the absence of cervical lymphadenopathy. Nasopharyngeal carcinoma (NPC) remains the primary consideration given its infiltrative growth pattern, deep parapharyngeal extension, and aggressive radiological appearance [17]. However, several imaging features in our case argue against this diagnosis, including the absence of cervical and retropharyngeal lymph node enlargement, lack of skull base marrow invasion, and no evidence of perineural tumor spread or dural involvement, which are typically associated with advanced NPC.

Lymphoma is another key differential diagnosis due to its potential to present as a bulky nasopharyngeal mass with restricted diffusion on MRI [16]. Nevertheless, lymphoma more commonly demonstrates homogeneous contrast enhancement and is frequently associated with multiple enlarged lymph nodes, features not observed in our patient.

Granulomatosis with polyangiitis may mimic neoplastic disease in the nasopharynx; however, it usually presents with destructive sinonasal changes, septal perforation, and systemic vasculitic manifestations, none of which were present in this case.

Invasive fungal infections represent an important consideration, particularly in aggressive-appearing nasopharyngeal lesions. However, they predominantly occur in immunocompromised patients and often show marked T2 hypointensity due to paramagnetic fungal elements, which was not demonstrated in our patient.

Sarcoidosis may involve the upper aerodigestive tract but typically presents with symmetric mucosal thickening and systemic lymphadenopathy, making it unlikely in this isolated presentation. Similarly, syphilitic involvement of the nasopharynx is exceedingly rare and usually associated with mucosal ulceration and systemic clinical signs, which were absent.

Overall, the combination of an aggressive radiological appearance in the absence of nodal disease, skull base invasion, or systemic features significantly broadened the differential diagnosis and underscored the diagnostic challenge, ultimately necessitating histopathological confirmation for definitive diagnosis.

The definitive diagnosis of nasopharyngeal tuberculosis relies on histopathological examination and microbiological analysis of biopsy specimens [18]. Multiple biopsies from different sites were performed to reliably exclude nasopharyngeal carcinoma or the rare possibility of a coexisting dual pathology, as previously reported in the literature [19].

In our patient, histopathological analysis revealed epithelioid granulomatous inflammation with multinucleated giant cells and central caseous necrosis, consistent with NPTB. Furthermore, identification of *M tuberculosis* by direct microscopy or culture on specific media may require up to 6 weeks and may yield false-negative results. In this context, molecular techniques such as PCR have emerged as valuable diagnostic tools, enabling earlier and more reliable detection, particularly when histopathological findings are inconclusive [19]. In our case, the combined histopathological and PCR findings confirmed the diagnosis of primary NPTB.

Management of extrapulmonary tuberculosis generally follows the same therapeutic principles as pulmonary tuberculosis, except in cases where a specific causative microorganism is identified or resistance to first-line antituberculous agents is demonstrated [20]. Standard treatment consists of multidrug antituberculous therapy combining rifampicin, isoniazid, and pyrazinamide during an initial intensive phase of 2 to 3 months, followed by a continuation phase of 4 to 6 months with isoniazid and rifampicin [19]. Reported recurrence rates are low, estimated at approximately 1%, and are mainly associated with the emergence of multidrug-resistant *M tuberculosis* strains [21].

The therapeutic response may also provide valuable diagnostic insight. Nasopharyngeal tuberculosis typically shows a favorable response to standard antituberculous therapy, with regression of both the primary lesion and associated lymphadenopathy [22]. In our case, multidrug antituberculous treatment resulted in a favorable clinical course, characterized by marked improvement in the patient’s general condition and complete resolution of headache symptoms. Follow-up flexible nasopharyngoscopy and imaging studies demonstrated complete resolution of the nasopharyngeal lesion, with no evidence of recurrence or newly developed abnormalities, confirming an excellent therapeutic response.

Nasopharyngeal tuberculosis should be considered in the differential diagnosis of nasopharyngeal masses, even in immunocompetent patients and in the absence of systemic symptoms, particularly when imaging findings appear aggressive or atypical. A multidisciplinary approach integrating clinical presentation, imaging features, histopathology, and molecular testing is crucial to prevent misdiagnosis and ensure appropriate management.

## Conclusion

Nasopharyngeal tuberculosis is a rare and often underrecognized condition that may closely mimic malignant nasopharyngeal tumors on imaging, particularly in the presence of atypical clinical and aggressive imaging features. This case emphasizes the importance of maintaining a high index of suspicion, even in immunocompetent patients without systemic symptoms. Accurate diagnosis relies on a multidisciplinary approach combining imaging, histopathology, and molecular testing, allowing timely treatment and an excellent prognosis.

## Patient consent

A written informed consent was obtained from the patient for the publication of this case report.
